# Anti-Biofilm and Anti-Quorum-Sensing Activity of *Inula* Extracts: A Strategy for Modulating *Chromobacterium violaceum* Virulence Factors

**DOI:** 10.3390/ph17050573

**Published:** 2024-04-30

**Authors:** Petya D. Dimitrova, Viktoria Ivanova, Antoaneta Trendafilova, Tsvetelina Paunova-Krasteva

**Affiliations:** 1 Stephan Angeloff Institute of Microbiology, Bulgarian Academy of Sciences, Akad. G. Bonchev Str. Bl. 26, 1113 Sofia, Bulgaria; pdimitrova998@gmail.com; 2 Institute of Organic Chemistry with Centre of Phytochemistry, Bulgarian Academy of Sciences, Acad. G. Bonchev Str., Bl. 9, 1113 Sofia, Bulgaria; viktoria.genova@orgchm.bas.bg (V.I.); antoaneta.trendafilova@orgchm.bas.bg (A.T.)

**Keywords:** biofilms, *Chromobacterium violaceum*, anti-biofilm, anti-quorum-sensing activity, *Inula* extracts, virulence factors, antimicrobial resistance

## Abstract

The formation of microbial biofilm is a self-organizing process among bacterial cells, regulated by quorum-sensing (QS) mechanisms, contributing to development of infections. These processes, either separately or in combination, significantly contribute to bacterial resistance to antibiotics and disinfectants. A novel approach to addressing the challenge of treating infections due to antibacterial resistance involves the use of plant metabolites. In recent years, there has been increasing recognition of different phytochemicals as potential modulators. In our study, we evaluated the synergistic effect of chloroform and methanol extracts from *Inula* species against key virulence factors, including biofilm formation, violacein production, and swarming motility. Each of the 11 examined plant extracts demonstrated the ability to reduce biofilms and pigment synthesis in *C. violaceum*. Two of the extracts from *I. britannica* exhibited significant anti-biofilm and anti-quorum-sensing effects with over 80% inhibition. Their inhibitory effect on violacein synthesis indicates their potential as anti-QS agents, likely attributed to their high concentration of terpenoids (triterpenoids, sesquiterpene lactones, and diterpenoids). Scanning electron microscopy revealed a notable reduction in biofilm biomass, along with changes in biofilm architecture and cell morphology. Additionally, fluorescence microscopy revealed the presence of metabolically inactive cells, indicating the potent activity of the extracts during treatment. These new findings underscore the effectiveness of the plant extracts from the genus *Inula* as potential anti-virulent agents against *C. violaceum*. They also propose a promising strategy for preventing or treating its biofilm formation.

## 1. Introduction

Bacteria having the ability to form attached communities surrounded by extracellular polymeric substances are known as biofilms. Adherent bacterial growth contributes to nosocomial and chronic infections, resulting in high mortality rates. Additionally, biofilms are associated with severe infections on medical implants such as prostheses, stands, catheters, and eye lenses, and biofouling of various surfaces [1]. According to the WHO, more than 80% of bacterial infections in developed countries are due to the ability of bacteria to form biofilms [2,3,4]. The structure of biofilms determines their resistance. The presence of several bacterial layers helps bacterial cohesion, and masks and neutralizes various external influences, including antibiotics. Also, the deposition of exopolysaccharide substances and their further formation in a polymer matrix supports the mechanical properties, stability, and protection of biofilms.

Today, it is well known that biofilm formation is under the control of a regulatory system for intercellular communication, known as “Quorum sensing” (QS). QS is a system of signals, receptors, and effectors through which bacterial intercellular communication takes place. It enables them to share information about the cell density in the environment and, in relation to this, to redirect the expression of some genes. As a result, bacteria collectively regulate their energy-intensive processes so that they are activated only when needed and with maximum efficiency. Other key mechanisms, such as virulence, gene expression, pathogenicity, and antibiotic resistance, are thus regulated [5,6]. The ability to interfere with QS signals and disrupt essential components, such as extracellular DNA (eDNA), proteins, and lipopolysaccharides, suppresses the formation of the polymer matrix [7] and virulence factors, inhibits cell adhesion and attachment, and ultimately leads to the blocking of the “QS-network” and biofilm inhibition [8].

*Chromobacterium violaceum* is a motile, free-living, saprophytic Gram-negative, facultative, deep-purple colony-forming anaerobic bacterium [6,9]. The color of its colonies is due to the release of the pigment violacein, encoded by the *vio* operon, whose expression is controlled by QS signals. *C. violaceum* is a saprophyte found mainly in soil and water with optimal growth development at 30–35 °C [10,11]. This bacterium is associated with a number of diseases in humans, including infections of the respiratory and gastrointestinal tracts, liver, meningitis, endocarditis, and sepsis [10]. *C. violaceum* is one of the most commonly used bacterial species in QS research and testing of potential naturally occurring QS inhibitors. Synthesis of the insoluble pigment violacein is readily detectable and quantifiable, allowing a quick and easy way to search for these potential inhibitors. Its ability to form biofilms is part of its virulence characteristics, which in turn increases its resistance to antibiotics, phagocytosis, and disinfectants, and enhances the initiation of infections in the host [11,12].

In order to address the escalating antibiotic resistance and dissemination of biofilms, it is important to explore novel strategies that target the formation of the extracellular matrix, biofilm adhesion and dispersion, and quorum-sensing signaling pathways with non-toxic and potent anti-biofilm agents. Substances with a proven good anti-biofilm effect must possess a unique structure that facilitates easy entry into the cell, influences QS signaling, and possesses synergism with other antibacterial agents. One such promising approach is the application of medicinal plant extracts. Their ability to possess various therapeutic activities—anticancer, antioxidant, antidiabetic, immunosuppressive, antifungal, antimalarial, antiviral, etc.—makes them a valuable and desirable resource for application [13]. To date, however, data on their application as anti-biofilm and anti-quorum inhibitors are missing or too scarce.

The genus *Inula* consists of approximately 100 species, distributed mainly in Asia and Europe. Several *Inula* spp., such as *Inula helenium* L. and *Inula britannica* L., as are used in traditional medicine throughout the world, although are more common in Traditional Chinese Medicine, and some of them are officially listed in various European pharmacopoeias [14]. Species from the genus *Inula* are characterized by high chemical diversity, and approximately 500 secondary metabolites have been identified to date, mainly terpenoids (sesquiterpene lactones) and phenolic compounds (phenolic acids and flavonoids) with relevant pharmacological activity [14,15,16,17,18]. The genus *Inula* is represented in the flora of Bulgaria by 10 species, most of which have been the subject of phytochemical studies in recent years. In our previous studies, it was found that the chloroform extracts of *I. britannica*, *I. bifrons* L., and *I. salicina* L. contain various triterpenoids [19] but differed in the content of other terpenoids. *I. britannica* was found to contain sesquiterpene lactones [20], and *I. bifrons* to contain sesquiterpene lactones and diterpene acids of the ent-kaurane type [21], while *I. salicina* was free of these secondary metabolites [19]. The methanol extracts of *I. britannica*, *I. bifrons* L., and *I. salicina* L. are rich in chlorogenic acid and dicaffeoyl esters of quinic acid [13,21]. The Bulgarian populations of *I. helenium* and *I. spiraeifolia* have not been studied phytochemically yet. A literature survey showed the presence of sesquiterpene lactones and mono- and dicaffeoyl esters of quinic acid in *I. helenium* aerial parts [17], while data regarding the chemical composition of *I. spiraeifolia* are lacking.

The present study aimed to evaluate the quorum-sensing modulating activity and biofilm inhibitory potential of various extracts from five species of the genus *Inula* against *C. violaceum*. Data on the chemical composition of plant extracts are reported; anti-biofilm and anti-quorum activity were evaluated; the ability to suppress motility was verified; the 3D surface architecture of biofilms was investigated by scanning electron microscopy; and the viability of bacterial biofilms was assessed by fluorescence microscopy.

## 2. Results and Discussion

### 2.1. Effects of Plant Extracts on Violacein Production in C. violaceum

The quorum-sensing system controls key mechanisms such as virulence, gene expression, pathogenicity, and antibiotic resistance [22]. This system plays a crucial role in bacterial pathogenicity [23] by regulating the activity of efflux pumps, thereby promoting resistance and tolerance to various antibiotics and disinfectants [24]. In pathogenic bacteria, the coordinated “behaviour and actions” are essential for a successful infection, which is achieved by the QS communication between them [25]. However, impaired quorum-sensing communication leads to decreased bacterial pathogenicity and the blockade of infection progression [5,6,23,26,27]. Anti-quorum-sensing agents aim not to kill bacteria, but rather to reduce their bacterial virulence [28].

Despite the extensive research focused on the anti-quorum-sensing properties of plant extracts, Bulgarian representatives of the genus *Inula* have yet to be tested and reported on. To evaluate their potential anti-QS effect, we employed tests to evaluate the synthesized pigment violacein. Violacein is a respiratory pigment involved in the regulation of tryptophan production. Its synthesis involves the expression of the *vio* operon, consisting of five enzyme-encoding genes (*vio*ABCDE), which are transcribed in one direction [10]. This process is controlled by the homologous system CviR/CviI (LuxR/LuxI) and the molecule N-hexanoyl-L-homoserine lactone [29,30], and is associated with biofilm formation. The ease of visualizing violacein production makes *C. violaceum* a valuable indicator for QS molecules and their inhibitors [31]. It has become evident that phytometabolites isolated from medicinal plants are effective in modulation of QS-controlled bacterial virulence factors, including biofilm formation, bioluminescence, motility, and pigment synthesis [6]. To investigate the impact of the plant extract treatments on violacein synthesis in the bio-reporter strain *C. violaceum*, the strain was cultivated in the presence of the extracts, followed by pigment extraction. Our primary objective was to assess the ability to suppress violacein production, potentially through inhibiting or controlling quorum-sensing mechanisms, using plant extracts.

The quantitative assays for violacein inhibition demonstrated inhibitory potential of over 50% for all tested extracts. The plant extract concentration of 250 μg/mL used in this study was chosen based on our previous experiments. Notably, significant reductions in pigment levels, reaching 83.59% ± 0.034 and 84.65% ± 0.089 at a concentration of 250 µg/mL, were observed in IBr1 and IBr1-SL, respectively (Table 1).

By comparison, the methanol extract is only more effective than the chlorophormic one in *I. spiraeifolia*. The high efficiency of chloroform extracts in the other *Inula* extracts may be attributed to the solvent used. When chloroform is used as the solvent, the extracts are rich in terpenoids [32]. It has been proven that these classes of compounds are highly effective against bacterial pathogens. They can exhibit antibacterial and anti-QS activity, as well as influence motility and biofilm formation [33]. Their potential for influence as quorum quenchers was demonstrated by three pentacyclic triterpenoids, glycyrrhetinic acid, ursolic acid, and betulinic acid, which are able to inhibit violacein and pyocyanin production in *C. violaceum* and *Pseudomonas aeruginosa* [34]. Moreover, the observed inhibitory effect of the enriched fraction of *I. britannica* may be attributed to its high concentration of sesquiterpene lactones. It is well known that sesquiterpene lactones possess a similar structure to AHLs, the signal molecules mediating QS in Gram-negative bacteria [35], and can negatively impact this process. Various sesquiterpene lactones isolated from *Polydora serratuloides* are recognized for their antimicrobial activity against Gram-positive bacteria. Furthermore, the action of these lactones has been confirmed to disrupt QS mechanisms in *C. violaceum* ATCC 12472. Combining these data with our findings, we can once again emphasize the contribution of sesquiterpene lactones to the suppression of quorum-sensing mechanisms in Gram-negative bacteria [36]. The introduction of this new strategy for suppressing quorum-sensing bacterial mechanisms is expected to be increasingly necessary in the future. Another type of sesquiterpene lactone, eudesmanolides, isolated from *Vernonia blumeoides*, also exhibited similar inhibitory effects on violacein production, as observed in our study in *C. violaceum*. Moreover, qualitative anti-quorum-sensing activity was demonstrated by agar diffusion double ring assays on two different bacterial biosensor systems, namely *C. violaceum* and *Agrobacterium tumefaciens* [35].

However, despite the lower activity of the methanolic extracts, they also exhibited significant effectiveness in inhibiting violacein production, ranging from 62% to 77%. A similar effect was observed with the methanol extract of *T. bellirica*, which inhibited violacein synthesis by 20–66% at concentration of 0.0625–0.5 mg/mL [37]. Our methanolic extracts inhibited violacein synthesis by approximately 70–80%. These data are supported by another study, in which methanolic extract from *A. annua* inhibited the pigment by ≥79% at a sub-MIC of 0.75 mg/mL [38]. When comparing our results with those available in the literature, it becomes evident that our applied concentrations of 250 μg/mL exhibit extremely promising effectiveness. This is particularly impressive when considering that the concentrations reported in previous studies were several times higher.

### 2.2. Effects of Plant Extracts on Biofilm Formation

In nature, bacteria frequently attach to surfaces and form multicellular consortia, protected by an extracellular polymeric matrix, commonly known as biofilms. Infections associated with biofilms represent a significant challenge for clinicians due to their high prevalence and their increasing antibiotic resistance. Thus, new strategies are needed to inhibit biofilm formation. To date, the effects of natural products from various sources have been tested, such as probiotics, honey, essential oils, and microbial metabolites, with the potential to affect biofilm formation. Individual studies have shown that some plant extracts obtained from medicinal plants have the ability to inhibit biofilm formation, as they can damage the cell membrane, block peptidoglycan synthesis, or interfere with the QS system [39].

Another important virulent factor of *C. violaceum*, besides pigment formation controlled by QS, is biofilm formation. The process of biofilm formation in *C. violaceum* is accompanied by an increase in virulence, through the manifestation of resistance not only to antibiotics and disinfectants, but also to phagocytic processes in the host [40].

**Figure 1:**
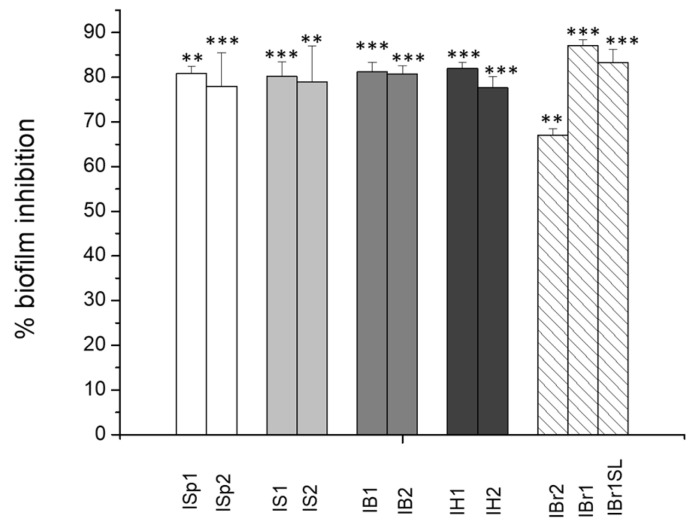
Effects of plant extracts on biofilm formation of *C. violaceum*. Statistically significant effects are indicated with asterisks ** *p* < 0.01; *** *p* < 0.001.

In our study, the effect of plant extracts at a concentration 250 µg/mL was evaluated by crystal violet staining and calculated as percentage of the control probe (Figure 1). The control probe demonstrated biofilm formation without plant substances, but with the addition of 2% DMSO. All of the tested extracts negatively affected the biofilm formation of *C. violaceum*, which is consistent with the anti-QS activity findings. The highest biofilm inhibition was detected by extracts isolated from *I. britannica*, with 87.12% ± 1.26 for IBr1 and 83.21% ± 3.09 for IBr1-SL, respectively (Figure 1). Values over 60%, but lower than 80%, were considered for IBr2, and the methanol extracts ISp2 isolated from *I. spiraeifolia*, IS2 from *I. salicina*, and IH2 from *I. helenium*. As observed in the anti-QS assay, the chloroform extracts were more effective. The presence of terpenoids as major components could influence bacterial attachment [41]. The quantitative results obtained from our study align with some literature findings regarding the influence of various plant extracts and their synthesized metabolites on biofilm formation in *C. violaceum*. For instance, it has been demonstrated that ethyl acetate extract from *Passiflora edulis* inhibited biofilm formation in a dose-dependent manner. Upon addition of 2 mg/mL of the extract, a 90.7% inhibition of biofilms was recorded [11]. In this case, the applied inhibitory concentrations are several times higher compared to those used in our study. Such research aims to achieve optimal effects at low treating doses, which is the outcome of our investigations.

### 2.3. Effects of Plant Extracts on Swarming Motility

Swarming motility is a collective and rapid mode of motility for bacteria, characterized by the formation of dynamic layers, and facilitates the swift invasion and successful colonization of tissues and nutrient-rich environments by bacterial cells [42]. A functional flagellum is required for this process to occur. The “swarming” cells undergo differentiation, accompanied by the expression of virulence determinants, contributing to the occupation of new niches [43]. In addition to increased virulence, these bacteria often display increased tolerance to various antibiotics. Thus, swarming motility is considered one of the most crucial virulence factors, alongside biofilm formation [44].

**Figure 2:**
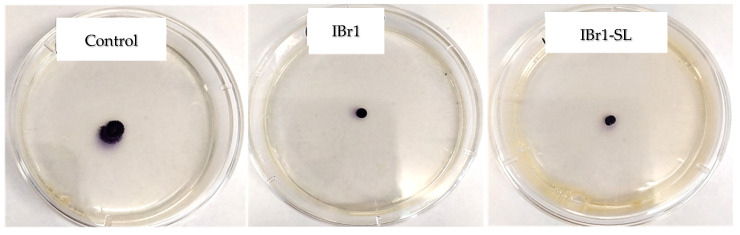
Effect of plant extracts from *I. britannica* on swarming motility of *C. violaceum*.

In this study, we assessed the motility of *C. violaceum* in the presence and absence of selected extracts. Our results indicate that both the chloroform extract (IBr1) and the enriched-fraction (IBr1-SL) from *I. britannica* inhibit swarming motility (Table 2). During the swarming process, branched paths were not observed; instead, a common movement front was evident (Figure 2).

When *C. violaceum* cells colonize different surfaces and niches, they tend to regroup, align parallel to each other, and “crawl” along the substrate. This process is known to be species-specific and is facilitated by exopolysaccharide synthesis [45]. Our findings align with several reports. For instance, one of them has shown that extracts from passion fruit (*P. edulis*) significantly inhibit swarming at concentrations ranging from 0.5 to 2 mg/mL [11]. Similarly, treatment with peppermint, thyme, and bergamot oil has been demonstrated to reduce the motility of *C. violaceum* by 40%, 45%, and 65%, respectively [46]. Furthermore, studies have shown that pure components such as apigenin and luteolin from *G. hypoleucum* DC exhibit a dose-dependent reduction in swarming motility [47].

In summary, both the IBr1 and IBr1-SL extracts derived from *I. britannica* demonstrated synergistic inhibitory effects on *C. violaceum*: an anti-biofilm effect, inhibition of violacein synthesis, and reduced swarming motility.

### 2.4. Effects of Plant Extracts on Biofilm Viability—Live/Dead Staining

**Figure 3:**
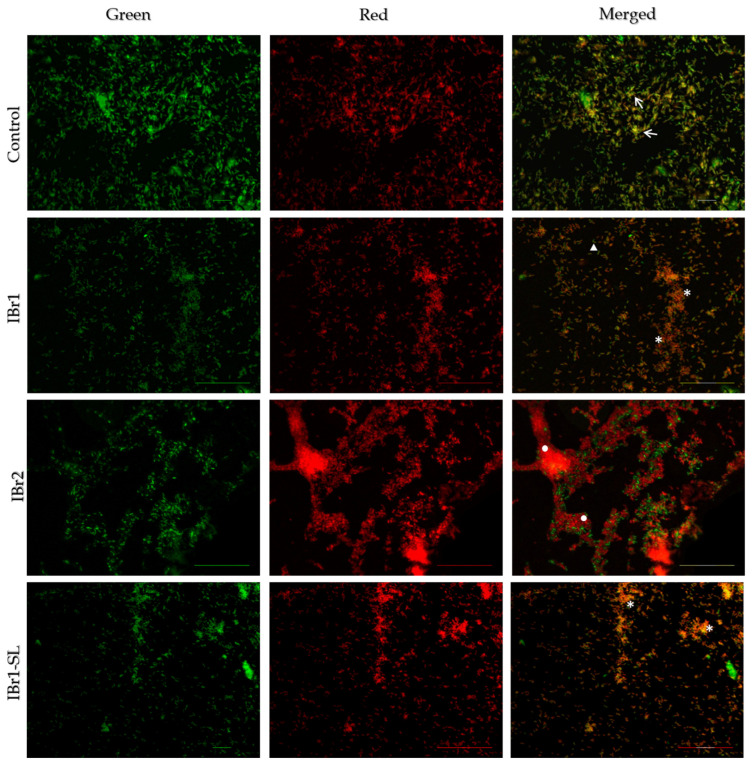
Biofilm viability of *C. violaceum* treated with *I. britannica* extracts (chloroform—IBr1, methanol—IBr2, enriched fraction with sesquiterpene lactones—IBr1-SL). White arrows—biofilm consortia consisting of viable cells; white star—biofilm consortia consisting of dead cells; white triangle—single live cells; white circle—dense and multi-layered biofilm. Bars = 20–50 μm.

The *I. britannica* extracts exhibited significant anti-biofilm and anti-QS activity. Nonetheless, it is crucial to assess how these extracts influence bacterial viability at a single-cell level and within the biofilm consortium. For this purpose, a Live/Dead fluorescent viability kit was applied that includes two components. Syto9, the first component, enters all bacterial cells and binds to their DNA, leading to the emission of green fluorescence. The second component, propidium iodide, selectively penetrates cells with altered permeability, binds to DNA, and emits red fluorescence. This dual staining approach enables the differentiation between live cells (green fluorescence) and dead cells (red fluorescence) within the biofilm structure. The fluorescence images of the control sample clearly revealed the presence of a relatively well-developed biofilm, primarily comprising intact, viable cells stained green (Figure 3). The biofilm appeared double-layered with the presence of local viable biofilm consortia (white arrow, Figure 3). Upon application of the IBr1 extract, a significant inhibitory effect was demonstrated on the cells within the formed biofilm consortia (white star, Figure 3). Accumulations of dead, broken-integrity bacterial cells were observed, alongside single live bacterial cells (white triangle, Figure 3). Similarly, the IBr1-SL extract displayed a significant inhibitory activity on both biofilm formation and cell viability. Biofilm clusters were rare, and the biofilm layer exhibited looseness, with predominantly metabolically inactive cells. In contrast, the fluorescence results of cells treated with IBr2 revealed distinct clustering and aggregation of biofilm layers, primarily composed of non-viable red bacterial cells. The architecture of the biofilm consortia appeared dense and multi-layered (white circle, Figure 3), with some surviving single cells in the upper layers of the mature biofilms.

Indeed, the fluorescence images provide compelling evidence that plant extracts can exert a significant influence on bacterial cells and the process of biofilm formation. Moreover, the impacts on cell viability further emphasize the multifaceted mechanisms through which these extracts can disrupt biofilm architecture and function. These findings corroborate the notion that plant extracts possess promising potential as agents for combating biofilm-associated infections.

### 2.5. Effects of Plant Extracts on Biofilm Morphology

**Figure 4:**
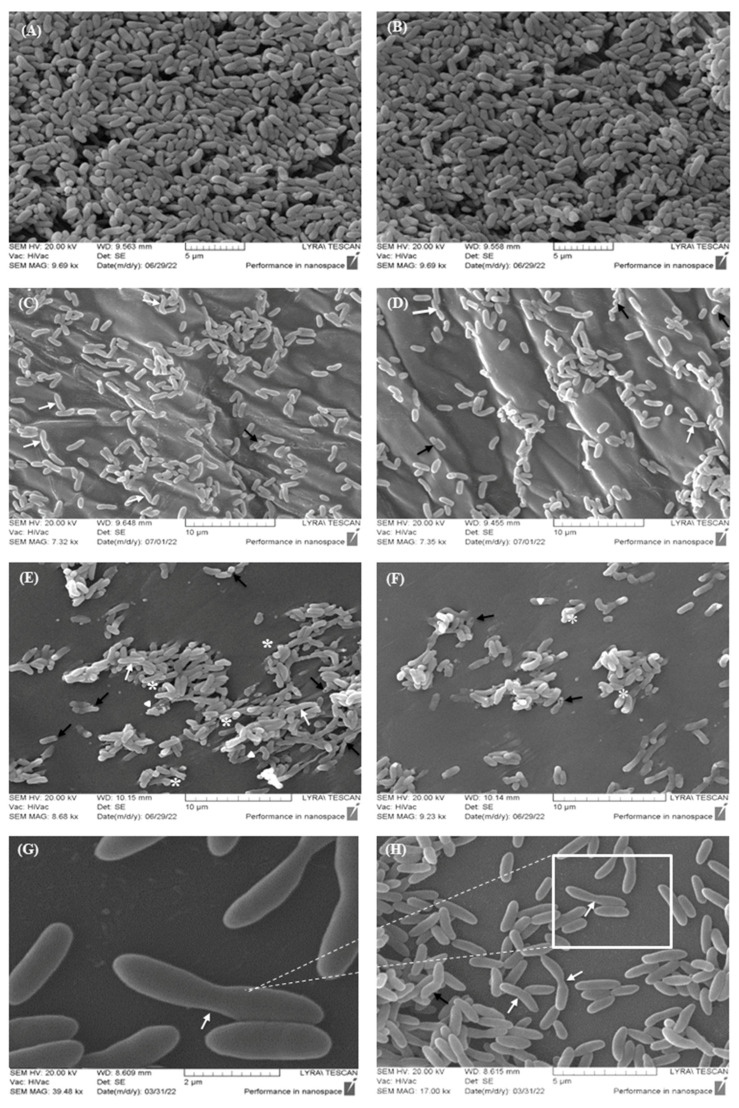
Scanning electron microscopy (SEM) demonstrating cell surface morphology and biofilm formation of *C. violaceum* treated with *I. britannica* extracts. (**A**,**B**) Control group—untreated cells with extracts; (**C**,**D**) treated cells with IBr1 extract; (**E**,**F**) treated cells with methanol extract—IBr2; (**G**,**H**) treated with the lactone-enriched fraction—IBr1-SL. White arrows—elongated cells; black arrows—invaginations; white star—swelling; white triangle—ruptured cells. Bars = 2–10 µm.

A series of SEM analyses were conducted to elucidate the structural and functional characteristics of biofilms treated with plant extracts. The most representative findings, exhibiting distinct effects on cellular surface morphology and reduction in the biofilm biomass, are presented in Figure 4.

In the control group, the cellular morphology of *C. violaceum* exhibited a smooth surface and normal sizes. There were no changes in the surface architecture associated with deformations and disruption of the cell permeability (Figure 4A,B). Furthermore, the morphology of the formed biofilm appeared flat and multilayered, with underlying layers consisting of several rows. However, in biofilms cultivated with the IBr1 extract, the cells appeared asymmetric with an abnormal shape, with some elongation in the area of the bacterial septa (white arrow, Figure 4C,D). Additionally, single cells with an asymmetrically and unevenly invaginated surface were found (black arrow). Similarly, cells treated with IBr2 extract exhibited morphological deformations (Figure 4E,F) characterized by invaginations on the cell surface (black arrow). The cell surface structure appeared deformed, with swelling areas (white star). Additionally, ruptured bacterial cells were observed (indicated by white triangles). A cohesive architecture and aggregation of the bacterial biofilm, confirmed also through fluorescence microscopy, were noted. This aggregation is likely due to the synthesis of EPSs (extracellular polymeric substances), in which the cells were immersed to varying extents. Indeed, this assumption is further supported by the SEM images of the control group, where cells were clearly differentiated with defined morphological contours. In contrast, in the cohesive biofilm areas treated with the extract, there was no clear differentiation between the cells. This lack of differentiation suggests that the extract may have disrupted the extracellular matrix or altered cell–cell interactions, leading to a loss of structural integrity within the biofilm. These observations support the notion that EPSs play a significant role in shaping the architecture of biofilms during maturation stages [48]. Additionally, the presence of chlorogenic acid in the methanol extract of *I. britannica* is likely the cause of the inhibitory activity, resulting in subsequent effects on biofilm formation in *C. violaceum*. Similar data have been reported not only for *C. violaceum* but also for *P. aeruginosa*, where chlorogenic acid affects not only biofilm formation but also swarming motility, protease and elastase activities, and rhamnolipid and pyocyanin production [49]. The SEM images taken upon treatment with the lactone-enriched fraction IBr1-SL revealed abnormally shaped bacterial cells, likely resulting from incomplete cell division. There was a certain elongation of cell size resembling filamentous forms, along with disruption in the processes of cell division and an inability to form bacterial septa (white arrow, Figure 4G,H). The biofilm structure appeared to be organized into single island formations, with loose areas consisting of only a few cells (Figure 4H). These findings indicate that IBr1-SL extract not only affects the structural characteristics of individual cells but also disrupts the process of biofilm formation. Additionally, the SEM images of the IBr1-SL treated biofilm align with the results from the CV assay, wherein one of the distinct inhibitions of the biofilm biomass was observed (83%) from *I. britannica* species.

The data obtained from the 3D biofilm microstructure analysis and the observed aberrant changes in the cells provide further confirmation of the synergistic action of the plant extracts. These extracts demonstrate both anti-biofilm and structural-determining effects, as evidenced by the alterations observed in the biofilm architecture and the individual cell morphology. These effects that were observed in combination with significant inhibition of pigment synthesis, biofilm formation, and motility, indicate the potential of these plant extracts as effective anti-biofilm agents.

## 3. Materials and Methods

### 3.1. Plant Material

Plant material was collected in full flowering stage from native populations in Bulgaria during 2017–2019: *Inula salicina* (41°53′18.76″ N 23°22′13.18″ E, SOM 176701)—from Rila Mt.; *I. bifrons* L. (41°42′18.75″ N 24°34′15.88″ E, SOM176699)—from Rhodopes Mts.; *I. ensifolia* L. (41°29′30.34″ N, 23°26′59.71″ E, SOM176700)—from Struma River Valley; *I. britannica* L. (42°35′32.96″ N, 23°22′7.88″ E, SOM 172474)—from Sofia region; *I. helenium* L. (42°27′34.37″ N, 23°13′35.83″ E)—from Verila Mt.; and *I. spiraeifolia* L. (41°45′37″ N, 23°09′15″ E, SOM 176695)—from Struma River Valley. Plant species were identified by Dr. Ina Aneva (Institute of Biodiversity and Ecosystem Research, Bulgarian Academy of Sciences). Voucher specimens (SOM) have been deposited with the Herbarium of the Institute of Biodiversity and Ecosystem Research, Bulgarian Academy of Sciences. The aerial parts were air-dried and kept in a dark and cool place until extraction.

### 3.2. Extraction

Air-dried and powdered plant material (10 g) was subsequently extracted with chloroform (200 mL, 3 times) and MeOH (200 mL, 3 times) at room temperature for 24 h each. The extracts were filtered and evaporated under reduced pressure to give corresponding chloroform and methanol extracts. A portion of the chloroform extract from *I. britannica* was further subjected to column chromatography on silica gel using CHCl_3_/acetone (10:1) to give the enriched sesquiterpene lactones fraction (TLC control) [20]. The extracts (Table 3) were kept at −20 °C prior biological assays.

### 3.3. Cultural Conditions of Bacterial Strain C. violaceum 30191

The bacterial strain used in the study, *C. violaceum* 30191, was obtained from the Leibniz-Institute DSMZ-German Collection of Microorganisms and Cell Cultures (DSMZ, Braunschweig, Germany). It was stored in 8% DMSO at −80 °C. Before each experiment, the strain was inoculated in Luria-Bertani Broth (LB)—1% Casein enzymic hydrolysate, 0.5% yeast extract, 1% NaCl, (HiMedia, Modautal, Germany), and maintained at 4 °C on Luria-Bertani Agar (LA)—1% Casein enzymic hydrolysate, 0.5% yeast extract, 1% NaCl, 0.15% agar (HiMedia, Modautal, Germany) slants. As a source of bacterial inoculum, before each experiment, the strain was incubated in LB, without shaking at 30 °C for 24 h.

### 3.4. Screening for Violacein Inhibition by Plant Extracts

The anti-quorum-sensing activity of the extracts was evaluated using the bioreporter strain *C. violaceum* DMSZ 30191. The inhibitory potential of the plant extracts on the production of violacein was assessed according to the methodology of Choo (2006) [50] with slight modifications. An overnight bacterial culture of the strain was suspended in fresh LB broth in the presence of the extracts at a concentration of 250 μg/mL and then incubated at 30 °C for 24 h. The control probe contained LB broth and the strain without the plant extracts. After the incubation period, the tubes were centrifuged at 13,000 rpm for 10 min. to precipitate the bacteria and any undissolved violacein. The supernatant was removed, and the cell pellet was resuspended in DMSO. To ensure complete dissolution of the violacein, the samples were vortexed and centrifuged for 10 min at 13,000 rpm to disintegrate the bacterial cells and extract the violacein. The violacein present in the supernatant was measured at 585 nm absorbance using an ELISA plate reader (LTEK INNO, Gyeonggi-do, Republic of Korea).

The violacein inhibition assays were calculated based on the following formula:

Violacein inhibition (%) = control OD 585 nm − test sample OD 585 nm/control OD 585 nm × 100

### 3.5. Screening for Biofilm Inhibition by Plant Extracts

To determine the anti-biofilm activity of plant extracts against *C. violaceum*, the protocol described by Soto et al. (2006) was used [51]. Biofilms were cultivated in M63 media with the following composition: 0.02 M KH_2_PO_4_, 0.04 M K_2_HPO_4_, 0.02 M (NH_4_)_2_SO_4_, 0.1 mM MgSO_4_, and 0.04 M glucose. A culture of the bacterial strain in a 1:100 ratio, diluted in M63 media overnight, was used as the starting inoculum for the biofilm experiments. Plant extracts with a final concentration of 250 µg/mL were loaded onto 96-well, U-bottomed polystyrene microtiter plates (Corning, Corning, NY, USA), followed by the addition of the diluted bacterial strain to a final volume of 150 µL. The control sample contained the bacterial inoculum diluted in M63 with 2% DMSO. Each sample was applied in six replicates. The plates were then incubated for 24 h at 30 °C under static conditions. After incubation, the wells with planktonic bacteria were washed three times with phosphate-buffered saline (PBS, pH 7.2), and the adherent bacteria were stained with 0.1% of aqueous crystal violet for 15 min. Finally, the wells were washed with PBS and solubilized with 70% ethanol. The optical density was measured at a wavelength of 570 nm using an ELISA plate reader (LTEK INNO, Gyeonggi-do, Republic of Korea).

### 3.6. Swarming Motility Assays

To conduct swarming motility assays, sterile plastic Petri dishes were prepared containing 0.6% LA supplemented with 0.2% (*w*/*v*) filter-sterilized glucose and 250 μg/mL final concentration of the plant extracts. LA agar without DMSO and plant extracts was used as a positive control to determine the normal motility. The sterile plastic agar plates were inoculated in the center with an overnight bacterial culture of *C. violaceum*. Subsequently, the plates were incubated for 24 h at 37 °C. Each sample was tested in duplicate, and the diameters of the swarming migration zones were measured and compared to the control sample.

### 3.7. Assessment of Biofilm Viability Using Live/Dead Staining

To access bacterial viability in the biofilms, they were cultivated on borosilicate sterilized cover glasses. Cultivation was performed in 24-well cell culture plates in which the sterile glasses were covered with a bacterial suspension containing 250 μg/mL plant extracts or 2% DMSO as a positive control probe. The biofilms were cultivated for 24 h at 37 °C. After treatment, the biofilms were washed and stained with the Live/Dead BacLight Bacterial Viability Kits (Invitrogen, Carlsbad, CA, USA) following the manufacturer’s procedure. Subsequently, the samples were mounted on glass slides using Fluoromount Mounting Medium (Sigma, New York, NY, USA). Observations were made using a confocal laser scanning microscope (CLSM) Nikon Eclipse TiU, at excitation wavelengths of 488 nm and 543 nm in epifluorescence mode with 60× oil PlanApo objective. Representative images were randomly taken from each sample using a CCD camera, Nikon DS-Fil (Melville, NY, USA). The images were processed using NIS-Elements software (Ver. 4.0) and the Icy bio-imaging program (Ver. GPLv3).

### 3.8. Scanning Electron Microscopy (SEM)

Morphological alterations of bacterial cell surface and biofilm formation in the presence of plant extracts were investigated using SEM. *C. violaceum* biofilms were incubated on sterile plastic pieces and treated as described above. After the treatment, samples were washed with PBS and fixed with 4% glutaraldehyde in 0.1 M Na cacodylate buffer (pH 7.2) and incubated for 2 h at 4 °C. After that, they were washed again in cacodylate buffer and post-fixed for 1 h using a solution of 1% OsO_4_ again at 4 °C. A dehydration procedure was performed in a graded ethanol series through 15 min time intervals. Finally, the samples (with duplicates per variant) were mounted on scanning electron microscopy holders and sputtered with gold using a vacuum evaporator (Edwards, Irvine, CA, USA). Observations were made on a Lyra/Tescan scanning electron microscope (TESCAN GROUP a.s., Brno, Czech Republic) with an accelerating voltage of 20 kV.

### 3.9. Statistical Analysis

To confirm the quantitative data of biofilm and violacein inhibitions, the statistical significance (** *p* < 0.01; *** *p* < 0.001, sample number *n* = 6) was calculated of all the tested samples versus the control probe, using one-way analysis of variance (ANOVA) and indicated with asterisks. The data are reported as means ± standard deviation (SD) using OriginPro 6.1 software.

## 4. Conclusions

In this study, various methodologies were employed to assess the efficacy of plant extracts from the genus *Inula* as potential anti-virulent agents. All 11 investigated plant extracts exhibited suppression of *C. violaceum* biofilms, with the extracts from *I. britannica* demonstrating particularly significant anti-biofilm activity. Furthermore, the extracts were found to suppress violacein synthesis, highlighting their potential as anti-quorum-sensing agents. The better activity of the chloroform extracts as anti-QS inhibitors during violacein synthesis may be attributed to their composition, which is rich in terpenoids (triterpenoids, sesquiterpene lactones, and diterpenoids). SEM observations revealed that the extracts could disrupt the bacterial cell wall, leading to cell death, a finding supported by fluorescence staining results. Notably, the plant extracts were not only effective against individual bacterial cells but also influenced biofilm formation and its 3D structure. As evidenced by SEM and fluorescence imaging, the extracts not only induced cell death but also reduced the biofilm biomass. The efficiency of the tested plant extracts against Gram-negative bacteria and biofilm consortia is crucial for understanding the potential properties of *Inula* extracts as new anti-biofilm and anti-quorum-sensing agents. These new findings lay the foundation for the development of new strategies for the prevention and treatment of biofilm-associated infections.

## Figures and Tables

**Table 1:** Effects of plant extracts on violacein synthesis.

Inhibition of Violacein Synthesis, %										
ISp1	ISp2	IS1	IS2	IB1	IB2	IH1	IH2	IBr1	IBr2	IBr1-SL
52.14 ± 0.09 ***	62.02 ± 0.15 ***	77.08 ± 0.06 ns	75.76 ± 0.08 ***	80.39 ± 0.06 ***	72.29 ± 0.11 ***	78.33 ± 0.09 ***	77.8 ± 0.11 **	83.59 ± 0.09 **	77.88 ± 0.06 ***	84.65 ± 0.03 ***

Statistically significant effects are indicated with asterisks ** *p* < 0.01; *** *p* < 0.001, ns—non significant.

**Table 2:** Diameter of the motility zones.

Sample	Zone of Motility (mm)	% Inhibition
Control	12 ± 1	
IBr1	3 ± 0.05	75%
IBr1-SL	4 ± 0.5	66.66%

**Table 3:** Plant extracts used in the present study.

Extract	Type of Extract	Plant
ISp1	Chloroform	*I. spiraeifolia*
ISp2	Methanol	
IS1	Chloroform	*I. salicina*
IS2	Methanol	
IB1	Chloroform	*I. bifrons*
IB2	Methanol	
IBr1	Chloroform	*I. britannica*
IBr2	Methanol	
IBr1-SL	Enriched fraction with sesquiterpene lactones	
IH1	Chloroform	*I. helenium*
IH2	Methanol	

## Data Availability

Data is contained within this article.
